# Short tandem repeat profiling via next-generation sequencing for cell line authentication

**DOI:** 10.1242/dmm.050150

**Published:** 2023-10-23

**Authors:** Yi-Hsien Chen, Jon P. Connelly, Colin Florian, Xiaoxia Cui, Shondra M. Pruett-Miller

**Affiliations:** ^1^ Genome Engineering & Stem Cell Center (GESC@MGI), Department of Genetics, Washington University School of Medicine, St Louis, MO 63110, USA; ^2^ Center for Advanced Genome Engineering (CAGE), Department of Cell and Molecular Biology, St. Jude Children's Research Hospital, Memphis, TN 38105, USA

**Keywords:** Next-generation sequencing, Short tandem repeat, Targeted deep sequencing, Cell line authentication, Capillary electrophoresis, Cell identity

## Abstract

Cell lines are indispensable models for modern biomedical research. A large part of their usefulness derives from the ability of a cell line to proliferate over multiple passages (often indefinitely), allowing multiple experiments to be performed. However, over time, cell line identity and purity can be compromised by human errors. Cross-contamination from other cell lines and complete misidentification are both possible. Routine cell line authentication is a necessary preventive measure and has become a requirement for many funding applications and publications. Short tandem repeat (STR) profiling is the most common method for cell line authentication and is usually carried out using standard polymerase chain reaction-capillary electrophoresis analysis (STR-CE). Here, we evaluated next-generation sequencing (NGS)-based STR profiling of human and mouse cell lines at 18 and 15 loci, respectively, in a high-throughput format. Using the Python program STRight, we demonstrate that NGS-based analysis (STR-NGS) is superior to standard STR-CE in terms of the ability to report the sequence context of repeat motifs, sensitivity and flexible multiplexing capability. STR-NGS is thus a valuable alternative for cell line authentication.

## Teaser

**Summary:** Evaluation of next-generation sequencing (NGS)-based short tandem repeat (STR) profiling of human and mouse cell lines in a high-throughput format revealed that STR-NGS is a valuable platform for human and mouse cell line authentication.

## INTRODUCTION

Since the introduction of short tandem repeats (STRs) as polymorphic DNA signatures (Puers et al., 1993), STR profiling has become the gold standard for identity confirmation in contemporary forensic science (Butler, 2007). STRs, also known as microsatellites or simple sequence repeats, are DNA segments containing core repeat units of two to six nucleotides that are scattered throughout the genome (Ellegren, 2004). In addition to the original 13 core STR loci (Budowle et al., 1997), seven more loci were included in the Combined DNA Index System (CODIS) that is used for forensics in the United States (Hares, 2015). These STR loci are highly polymorphic, genetically unlinked, and offer powerful and accurate individual identification.

Human and mouse cell lines are important research models for mechanistic studies, target identification and therapeutic development. However, cell cultures are at risk of misidentification owing to human errors and cross-contamination from other cell lines. Examples of misidentified cell lines jeopardizing scientific research continue to grow in number (Nardone, 2007), demonstrating the urgent need for frequent cell line authentication. As a result, cell line authentication is now required by an increasing number of journals prior to publication, as well as for grant applications (Almeida et al., 2016). A method that is sensitive, high throughput and economical is highly desirable.

The framework of using STRs for the authentication of human cell lines was first introduced in 2010 (Barallon et al., 2010). To date, many thousands of human cell line STR profiles are available, reflecting the unique donors from whom they were originally derived (Novroski et al., 2016). The American Tissue Culture Collection (ATCC) has published standard guidelines that recommend the use of at least eight STR loci (TH01, TPOX, vWA, CSF1PO, D16S539, D7S820, D13S317 and D5S8181, plus Amelogenin for gender identification) for human cell line authentication (ASN-0002). Moreover, recent studies report several additional STR loci that may be used to authenticate mouse cell lines (Almeida et al., 2019, 2014).

Currently, STR profiling is predominantly performed by resolving multiplexed, fluorescently labeled polymerase chain reaction (PCR) amplicons using capillary electrophoresis (Deforce et al., 1998). However, loci of the same size but with different sequences cannot be distinguished using this conventional method. The full sequences and nucleotide variations found in STR loci provide additional data that aid in identification. The conventional STR method also requires access to a specialized instrument, a genetic analyzer, which has limited potential for further improving sensitivity or throughput. With continuous technical improvements, NGS has become an attractive alternative for STR profiling. Different NGS platforms, such as Roche/454, Ion torrent and Illumina systems, have proven capable of sequencing the majority of STR loci in forensic science (Fordyce et al., 2015; Mikkelsen et al., 2014; Van Neste et al., 2014). NGS-based STR (STR-NGS) profiling has several advantages over the conventional method, including high throughput, low cost when running many samples, flexibility with which STR loci to include, quantitative measurements for mixed samples and high resolution for single-nucleotide polymorphisms (SNPs) (Bornman et al., 2012; Shin et al., 2017). This method has yet to be applied to cell line authentication.

In the present study, we evaluate the accuracy and sensitivity of the Illumina MiSeq platform for STR profiling of human and mouse cell lines, and demonstrate that the method is valid and scalable for routine quality control of human and mouse lines used in biomedical research.

## RESULTS

### NGS optimization for human STR profiles

**Fig. 1.**
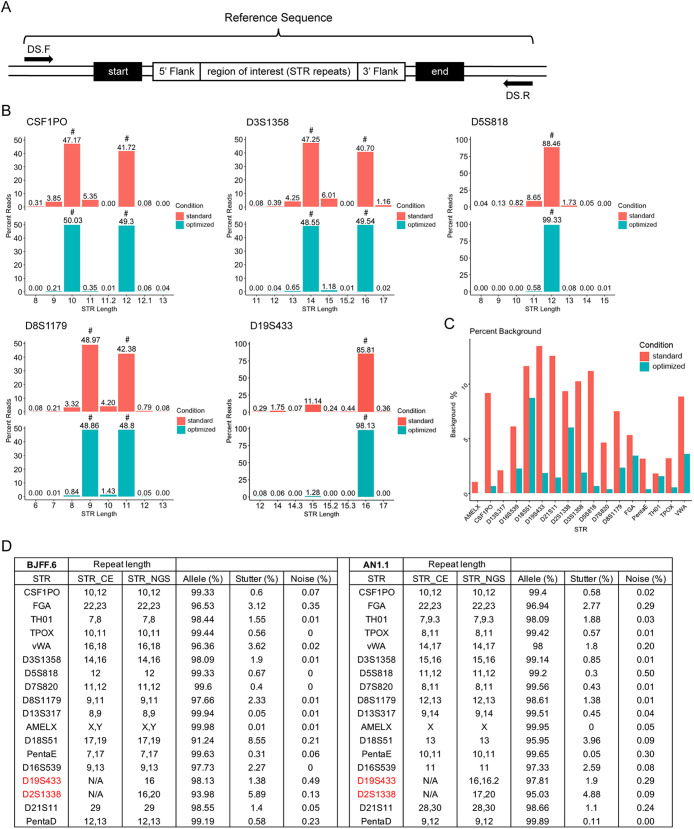
**STR-NGS optimization and performance.** (A) Schematic representation of a short tandem repeat (STR) locus with STR repeats, flanking regions at the start and end of the repeat region, and a targeted deep sequencing (DS) primer pair. Sequence from ‘5′ Flank’ to ‘3′ Flank’ is used for repeat length analysis. (B) Allele and stutter frequencies for five representative STR loci calculated as the percentage of the parent allele reads. Verified STRs are indicated by a ‘#’ above the bar plot. Standard and optimized conditions are MyTaq and SuperFi+tetramethylammonium oxalate (TMAO), respectively. (C) Total background (stutter+noise) in all STR loci in the comparison between standard and optimized PCR conditions. (D) STR profile for each locus examined by PCR-capillary electrophoresis (CE)- and next-generation sequencing (NGS)-based methods in two diploid induced pluripotent stem cell (iPSC) lines. Repeat lengths containing a decimal point indicate that an additional partial repeat is present in the locus. Red text indicates STRs that differed between STR-CE and STR-NGS. N/A, not available with STR-CE.

We developed an STR-NGS profiling method that amplifies and analyzes common human and mouse STR loci for repeat length. Primers designed to each STR locus amplify a region of interest (ROI) containing the STR repeats flanked by left and right consensus sequences (Fig. 1A). Our NGS library construction is a two-step process. PCR1 amplifies the region of interest and adds partial Illumina adaptors to the amplicons. PCR2 is the indexing PCR and adds unique identifiers to each sample. To improve NGS read quality, we optimized the PCR1 reaction at each locus. First, different primer sets were tested for amplification efficiency and specificity, and the final primer pairs for the 17 CODIS STR loci and sex (AMEL) locus in this study are listed in Table S1. Second, we evaluated different DNA polymerases, including several high-fidelity DNA polymerases – AccuPrime Taq DNA polymerase high fidelity from Thermo Fisher Scientific, Platinum SuperFi PCR Master Mix from Thermo Fisher Scientific and Titanium Taq DNA polymerase from Takara. MyTaq from Bioline is a low-cost, high-performance polymerase and was used as a standard for comparison. We also tested and optimized the quantity of genomic DNA sample input. We found that Platinum SuperFi PCR mix produced higher PCR amplicon yield and superior accuracy for this application (data not shown). SuperFi was used for subsequent tests and studies. As reported previously, we found that the quantity of input template DNA has a substantial effect on the outcome of PCR reactions (Lorenz, 2012). The optimal DNA amount in our assays is 70-140 ng per reaction, roughly equivalent to 10-20,000 cells (Fig. S1A,B). Low amplification was observed with input cell numbers lower than this range (data not shown). PCR supplements have been shown to be effective in improving yields of specific PCR products at difficult loci (Lorenz, 2012). Given the presence of homopolymer stretches at many STR loci (Gettings et al., 2015), poor PCR amplification can result in higher error rates in NGS. Two types of errors that can occur are stutters and noise. We define stutters as sequence reads that contain a number of STR repeat lengths that are smaller or larger (predominantly one repeat shorter) than the predominant allele and with a frequency of less than 10%. Noise is defined as reads that have partial repeats, are likely to be the product of sequencing errors or off-target amplification, and occur at low percentages (generally less than 5% of total reads). Several tetramethylammonium (TMA) derivatives have been shown to increase the specificity of PCR and improve the yield of amplification products (DiLella and Woo, 1987; Kovarova and Draber, 2000). We tested two TMA derivatives, TMA chloride (TMAC) and TMA oxalate (TMAO). TMAC addition in PCRs resulted in modest improvement in reducing background in most STR loci compared to PCR with no additive (data not shown), whereas addition of TMAO to PCR reactions increased the yield of specific PCR products (Fig. S1A,B). Therefore, SuperFi+TMAO was used for PCR1 in subsequent studies.

We also optimized the indexing reaction (PCR2). Dual indexing has been proven to increase the accuracy of multiplex sequencing and throughput on the NGS platform (Kircher et al., 2012). High-fidelity polymerases, such as SuperFi, are effective at reducing errors introduced in the indexing reaction (Fig. S1B). Five representative STR loci are highlighted in comparison between standard and improved PCR conditions (Fig. 1B), and overall background improvement was observed in all loci (Fig. 1C). Notably, noise and stutter decreased for all STRs with the improved PCR conditions. Moreover, it should be noted that amplification efficiency varies at different loci, as measured by total NGS reads at each locus (Fig. S1C).

### STR-NGS versus STR-CE

We next compared the results of STR-NGS using our optimized conditions with those obtained using PCR-capillary electrophoresis (CE)-based STR (STR-CE), conducted by Cell Line Genetics (Madison, WI, USA), for two diploid induced pluripotent stem cell (iPSC) lines. The two sets of data are highly in agreement (Fig. 1D). In both cell lines, noise, stutter and allele percentages are shown for all 18 STR loci (Fig. 1D). Among all STR loci, 91-99% of the reads from STR-NGS corresponded to parent alleles, and noise comprised less than 1% of the total reads. The stutters ranged from 0 to 3.6%, except D18S51 and D2S1338, which had higher stutter percentages. Together, these results demonstrate that STR-NGS identifies human cell lines as accurately as the standard STR-CE method.

### Sensitivity and multiplexing capability of STR-NGS

**Fig. 2.**
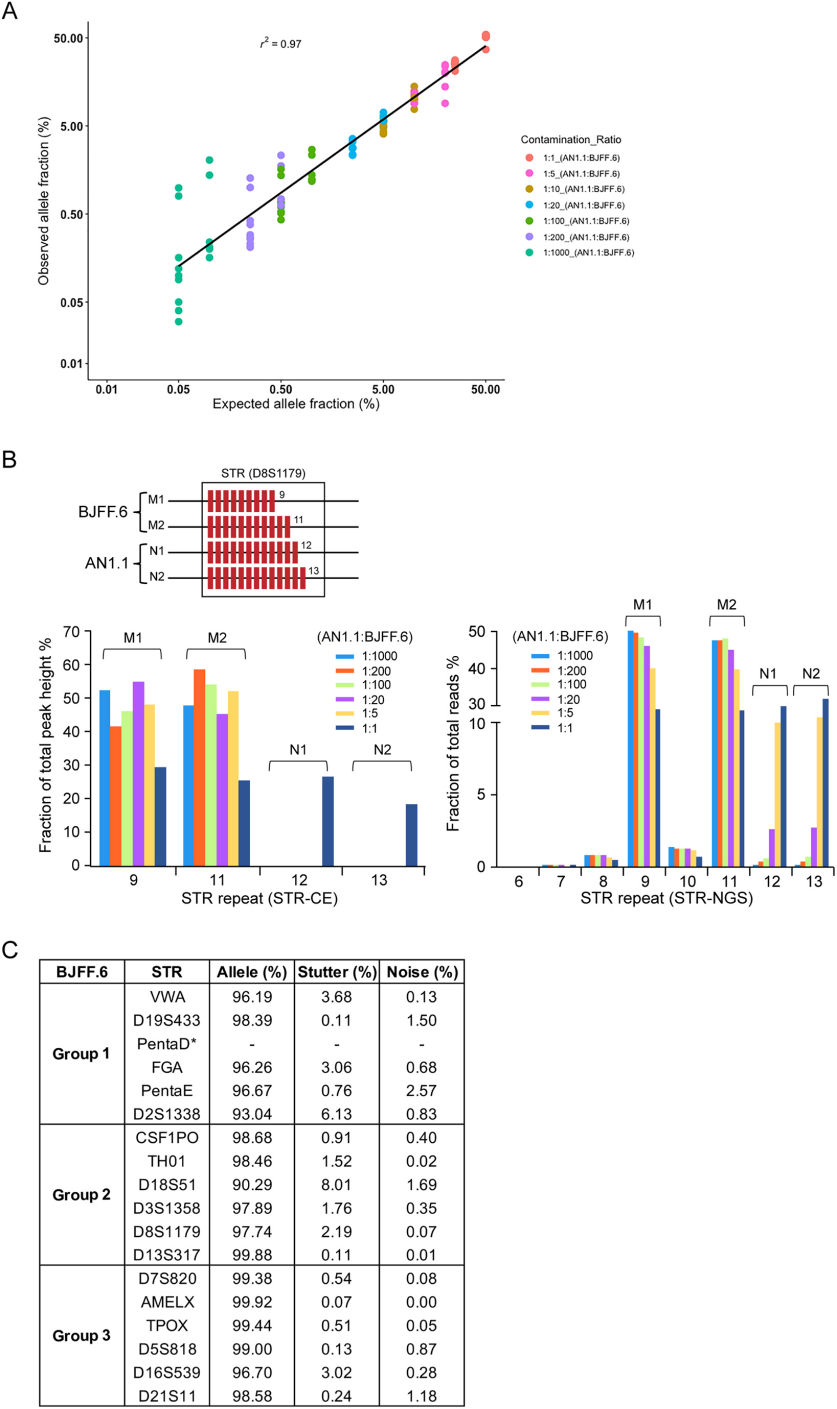
**STR-NGS sensitivity on a mixed sample and optimized multiplexed conditions.** (A) Observed allele fractions of informative STRs repeats are plotted against the expected ratio for given mixtures of two genomic samples. AN1.1 gDNA was diluted into BJFF.6 gDNA in a ratio of 1:1 to 1:1000. Expected allele fractions for diluted AN1.1 STRs correlate well with observed allele fractions. (B) A mixture of two diploid cell lines was analyzed for STR D8S1179. ‘M’ and ‘N’ alleles indicate genotypes from the major and minor components, respectively. Bar graphs shows percentage read counts for each STR repeat of both cell lines in different mixture ratios. (C) STR profile for each locus examined using multiplexed STR-NGS in BJFF.6 iPSC cells. The asterisk indicates a dropout allele.

Early detection of cross-contamination in a given culture is an important application of STR-NGS. We evaluated STR-NGS sensitivity in detecting a contaminating cell line sample among varying mixed ratios of BJFF.6 and AN1.1 cells, where AN1.1 represented the minor component of the mixtures. These two lines share STRs of the same length at several loci; therefore, we focused on unique repeats found in AN1.1. We found that the AN1.1 fractions by read count correlate well with the expected ratios (Fig. 2A). Next, we examined the D8S1179 locus specifically for its distinct repeat lengths in two cell lines. The AN1.1 cell line has STR repeat lengths that are one to two repeats longer than the BJFF.6 repeats, which allow them to be differentiated from stutters (Fig. 1D). Given that the major stutter of D8S1179 is 4 bp shorter than the parent allele, it provided a good opportunity to identify the minor alleles from AN1.1. We found that although STR-CE could only detect contamination levels of a 1:1 (50%) for D8S1179 to 1:5 (20%) ratio for some other STRs (Fig. 2B, bottom left and Table S3, respectively), STR-NGS could detect a minor contamination level as low as 1:200 within a sample (Fig. 2B, bottom right).

To reduce the cost and labor of the STR-NGS assay, we explored the feasibility of multiplexing the PCR reactions. We grouped different numbers of STR loci for multiplexed PCRs. Up to six loci can be amplified in one PCR reaction without compromising stutter and noise levels (Fig. 2C; Fig. S2). Stutter levels for D18S51 and D2S1338 remained higher than for other loci regardless of PCR conditions, and dropout of the PentaD STR was observed during multiplexing reactions. Overall, we were able to obtain satisfactory percentages of clean reads for all loci except for PentaD and conclude that STR-NGS in multiplexed format is as reliable as STR-NGS using individual PCR reactions.

### STR-NGS in mouse cell line authentication

**Fig. 3.**
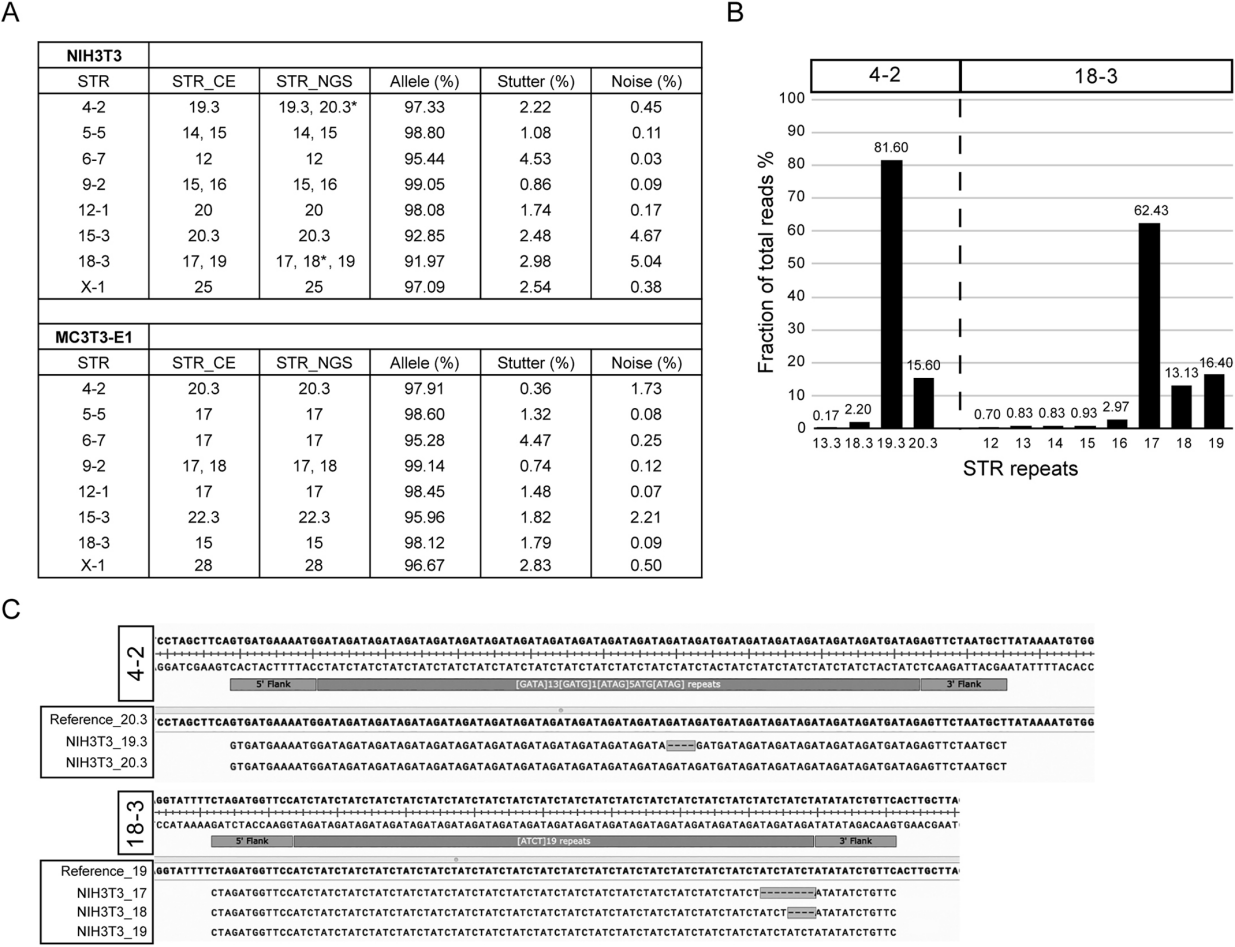
**STR-NGS in mouse cell lines.** (A) STR repeat length profiles in two mouse cell lines (NIH3T3 and MC3T3-E1) show allele, stutter and noise fractions of total parent allele reads. The asterisk indicates the difference in STR repeat length between reference and STR-NGS. (B,C) The frequencies of different stutters and repeat lengths (B), and sequence context of different STR repeat lengths (C) in two STR loci (4-2 and 18-3) are different between reference and STR-NGS in NIH3T3 cells. Repeat lengths containing a decimal point indicate that a partial repeat is present in predominant read length.

With great effort from the scientific community, STRs have been identified that allow authentication of mouse cell lines from different mouse strains (Almeida et al., 2019, 2014). To determine whether STR-NGS could be used for mouse cell line authentication, we performed STR-NGS on two previously reported mouse cell lines, MC3T3-E1 and NIH3T3, derived from different mouse strains (Almeida et al., 2014). First, both cell lines were used to optimize PCR performance; optimized primer sets are listed in Table S2. For MC3T3-E1, all loci matched the published STR-CE results (Fig. 3A). Of note, the STRs at loci 6-7 and 15-3 had higher stutter or noise percentages, respectively, than the other STRs assayed. Nonetheless, the combined stutter and noise for all STRs for MC3T3-E1 was less than 5% of the total reads. However, for NIH3T3 cells, minor fractions of different repeat lengths were observed at loci 4-2 and 18-3 (Fig. 3A,B).

To investigate the reason for the discrepancy in STR repeat length in the NIH3T3 cells, we compared the read percentages and sequencing content of different repeat lengths in STR loci 4-2 and 18-3. Stutter products in most loci usually comprise less than 5% of the total reads in both human and mouse samples (Fig. 1C and Fig. 3A). The read percentages for additional lengths were 15.60% for 4-2 and 13.13% for 18-3, which is unlikely to be the result of suboptimal assay conditions. One of the main advantages of the NGS-based method is that the raw data contain the actual sequence of a given locus. We examined the STR-containing reads with complete microsatellite sequences. At STR 4-2, our finding is consistent with results from a recent study (Almeida et al., 2019), which show 19.3 and 20.3 STR repeats for NIH3T3 cells (Fig. 3C). At STR 18-3, we observed reads corresponding to three repeat lengths – 17, 18 and 19. We hypothesized that the pool of immortalized NIH3T3 cells might contain two clonal populations harboring different repeat lengths at STR 18-3. To verify that the 18 repeats did not come from stutter products of 17 and 19, we performed single-cell sorting and analyzed single-cell clones from parental NIH3T3 cells. Three of the five independent clones carried 17 and 18 repeats at the locus (Table 1; Fig. S3). Another advantage of STR-NGS is that some information can be inferred based on the number of sequence reads for each repeat length. For example, all five clones show an allelic ratio of ∼2:1 for either the 17:18 or 17:19 repeats (Fig. S3), which suggests that there are three copies of this locus.

**Table 1.**
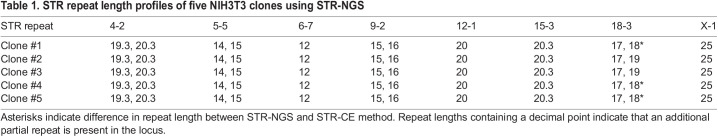
STR repeat length profiles of five NIH3T3 clones using STR-NGS

Next, we expanded the number of total mouse STR loci to 15 based on a previous study (Almeida et al., 2019) and compared the results between STR-CE and STR-NGS using the three mouse cell lines used in the previous study – NIH3T3, CT26 and 4T1. In all three lines, STR-CE and STR-NGS results matched at 12 of 15 loci tested (Table 2). To assess the differences among the three cell lines, we performed sequencing alignment with the data from parental cells and single-cell clones. In NIH3T3 cells, STR-NGS was able to detect the alleles from different subpopulations present in the pool (Fig. S4). Three unique subpopulations were identified by the presence of three different repeat lengths in STR locus 11-2 (Fig. S4A). To investigate further, we isolated, expanded and analyzed 12 single-cell clones. Most clones were represented by repeat lengths of 15 and 17. However, five of 12 clones presented with 15, 16, 17 or 14, 15, 17 repeat lengths (Fig. S4B). A unique clonal population was also observed in 4T1 cells (Fig. S5) in which the 17-2 locus differed between STR-NGS and STR-CE. STR-CE only showed a repeat length of 15 in 4T1 cells compared to repeats of 14 and 15 given by STR-NGS (Table 2). Given that the stutter for the 17-2 locus was below 6% in NIH3T3 and CT26 cell lines (Table 2), we believe that a repeat of length 14 exists and is from the minor allele contributor (Fig. S5B). Together, these data demonstrate that STR-NGS, compared to the traditional STR-CE method, offers great accuracy on allele calling and is a valuable alternative for mouse cell line authentication.

**Table 2.**
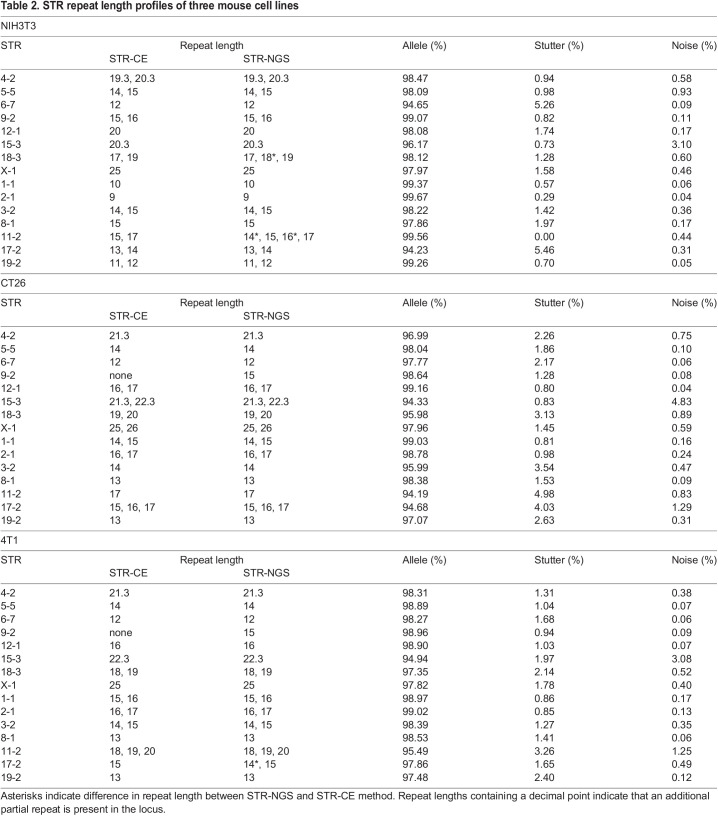
STR repeat length profiles of three mouse cell lines

## DISCUSSION

STR profiling is the gold standard for human identity testing (Butler, 2007) and cell line authentication (Barallon et al., 2010), given the uniqueness of each individual and relatively easy PCR-based assay. STR-CE is widely used for separating STR markers based on size distribution; however, it has limited sensitivity and does not provide the STR sequence identity or context. Although NGS has been implemented for human identification in forensic genetics, retroactive analysis of whole-genome sequencing and long-read sequencing (Bornman et al., 2012; Børsting and Morling, 2015; Valle-Silva et al., 2022), cell line authentication has been limited to STR-CE. Because of heterogeneity within many cell lines, this lack of sensitivity could lead to cell line misidentification or delayed identification of cross-contamination. Here, we present a simple and robust NGS-based solution for human and mouse cell line authentication using optimized amplification conditions for each STR locus and a novel STR analysis program called STRight.

To develop a robust STR-NGS workflow, we first evaluated different DNA polymerases, cell number, indexing PCR conditions and additives on STR loci amplification. After optimization, we achieved over 96% allele-specific signals at most loci. Next, we compared the results from STR-CE and STR-NGS workflows on two diploid human iPSC lines. Overall, the STR-NGS and STR-CE results matched. Moreover, except for D18S51 and D2S1338, the average range of stutter and noise percentage observed per STR locus is below 5% and 1%, respectively, for STR-NGS, compared to a stutter ratio of 20% for STR-CE, which is often used for initial filtering (Brookes et al., 2012; Mikkelsen et al., 2014; Novroski et al., 2016). Sequencing errors were minimal in most STR loci, with D18S51 and D2S1338 the obvious underperformers in the current protocol. Although both loci were amplified efficiently, a higher proportion of the associated reads contained errors in the ROI that confounded the results (data not shown). It is unclear at which point the errors are introduced, but a longer ROI and sequence context could be potential factors contributing to the stutter percentage and sequencing errors.

A previous study showed that, in mixed samples, sequences from the minor fraction (down to 1%) were detectable by NGS (Fordyce et al., 2015). In contrast, STR-CE has been reported to have a general detection limit for the minor contributor in 1:10 mixtures (Westen et al., 2009). In our comparison, we were only able to detect a contaminating cell line at 1:1 or 1:5 mixtures when using STR-CE. In this study, we show that STR-NGS is sensitive in detecting a minor contributor among a series of DNA mixtures down to 1:200 (Fig. 2B).

Multiplexed PCRs for STR typing are both time and cost effective. Indeed, STR alleles are routinely analyzed by multiplexed PCRs followed by CE-based analysis (Butler, 2006, 2007). However, large variations in target loci lengths result in a bias towards loci with shorter amplicons and lead to an imbalanced locus coverage (Van Neste et al., 2012). Although up to four or ten multiplexed PCRs for STR sequencing have been demonstrated for 454 or Ion Torrent sequencing platforms, respectively, high noise ratios and poor performance on allele calling have limited their utility (Butler, 2007; Fordyce et al., 2015). In this study, we demonstrate that our optimized workflow for STR-NGS with TMAO additive is capable of multiplexing six STR loci, with less than 5% stutters of total reads in 15 of 17 total human STR loci. Moreover, we recommend that underperforming loci, such as PentaD, are avoided in a multiplexed PCR setting. A careful selection of loci and further PCR multiplex optimization could further improve the utility of the STR-NGS approach.

Unlike STR forensic analysis, STR analysis is more complicated in human cell lines owing to the heterogeneity and aneuploidy present in many immortalized and transformed lines. Indeed, STR profiles for a given human cell line from different sources can even vary, and genetic variation within cell lines has been well documented (Pourquier and Azzi, 2019). For example, several different STR lengths are recorded for K562 cells depending on the source (https://www.cellosaurus.org/CVCL_0004). The need for cell line authentication in mouse cell lines has also risen in recent years (Almeida et al., 2019, 2014), so much so that the ATCC is now offering mouse STR profiling as a service. In this study, we demonstrate, for the first time to our knowledge, that STR-NGS can be used in mouse cell line authentication. In addition to generating correct genotypes, STR-NGS data resulted in the identification of subrepeat variants from subpopulations of NIH3T3 cells that were undetected by the STR-CE method (Almeida et al., 2019, 2014). In contrast to fragment length analysis by STR-CE, sequencing compositions in the ROI by STR-NGS provide additional detail for better interpretation of STR variation. The main limitation of using STRight is the required access to a NGS platform and the associated cost of performing targeted deep sequencing. However, STR-NGS can be less expensive than STR-CE if a large enough number of samples is performed in one sequencing run. Additionally, sequencing technology and costs are continuously improving, allowing greater access in both academic and industry settings.

Here, we developed a simple workflow that offers a reliable solution for genotyping the most frequently used STR loci in human and mouse cell lines (Tables 3 and 4) without the need for a specialized instrument made for STR. Multiplexing reactions provide the flexibility to run many samples at once and reduce both time and cost for the operation. STR-NGS improves allele calling efficiency and detection sensitivity, and allows better differentiation of artifacts from minor contributors than the standard STR-CE method. STR-NGS can be easily used to authenticate cell lines as well as sensitively detect cross-contamination.

**Table 3.**
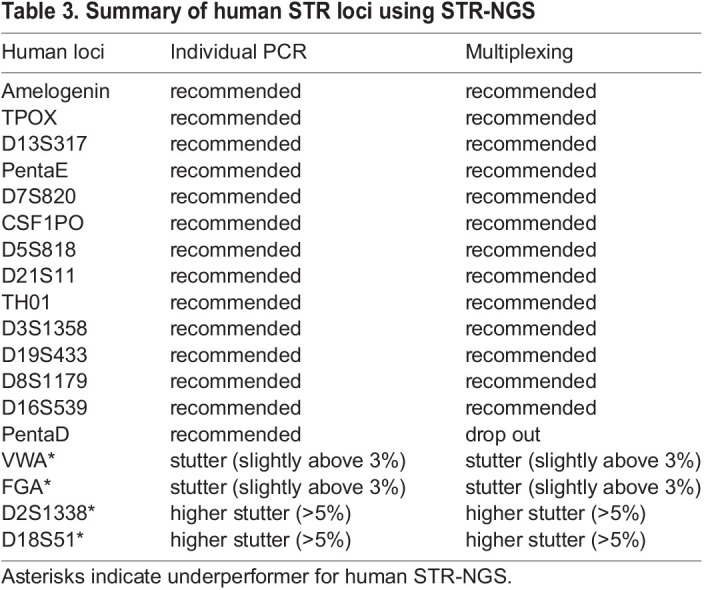
Summary of human STR loci using STR-NGS

**Table 4.**
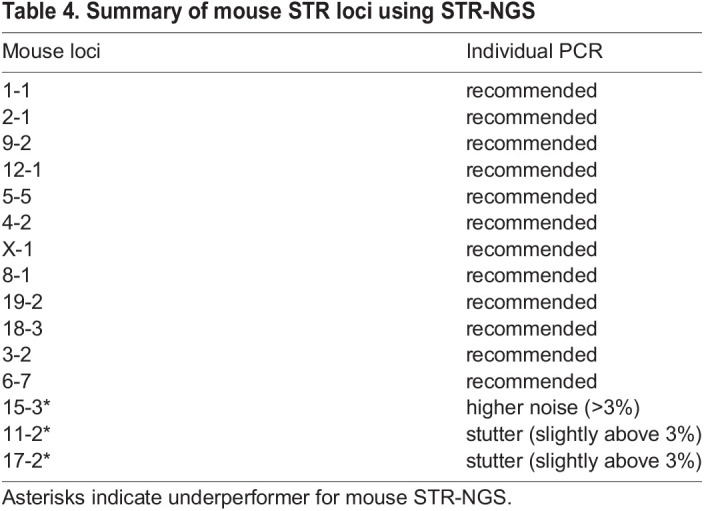
Summary of mouse STR loci using STR-NGS

## MATERIALS AND METHODS

### Control samples and cell lines

Two human iPSC lines, BJFF.6 from a male donor and AN1.1 from a female donor, were generated at the Genome Engineering & Stem Cell Center (GESC) at Washington University, St Louis, MO, USA. iPSCs were maintained on Matrigel (Corning)-coated plates in Stem-Flex medium (Thermo Fisher Scientific). In addition, four mouse cell lines – NIH3T3, MC3T3-E1, CT26 and 4T1 – were obtained from the ATCC and cultured in the ATCC-recommended media. All cell lines were grown in a humidified incubator set at 5% CO_2_ and 37°C.

### Extraction and quantification of DNA

DNA was extracted using a crude DNA extraction buffer (10 mM Tris-HCl pH8.0, 2 mM EDTA, 0.2% Triton X-100 and 200 µg/ml proteinase K) or a DNA Blood Mini kit (Qiagen) following the manufacturer's instructions. DNA was quantified using a NanoDrop One spectrophotometer (Thermo Fisher Scientific).

### STR loci and primer design

Eighteen STR loci (CSF1PO, D13S317, D16S539, D18S51, D19S433, D2S1338, D21S11, D3S1358, D5S818, D7S820, D8S1179, FGA, PentaD, PentaE, TH01, TPOX, vWA and Amelogenin) recommended by the ATCC and the CODIS were used for human cell line profiling. Fifteen STR loci (18-3, 4-2, 5-5, 6-7, 9-2, 12-1, 15-3, X-1, 1-1, 2-1, 3-2, 8-1, 11-2, 17-2 and 19-2) were used for mouse cell line STR profiling (Almeida et al., 2019, 2014). STR reference sequences were obtained from STRBase or Almeida et al. (2019, 2014) for human and mouse genomes, respectively, and used to structure STRight analysis. The Primer Blast design tool from NCBI was used to design PCR primers flanking STR regions based on the reference sequences from STRBase (Ruitberg et al., 2001) for human loci and two recent studies (Almeida et al., 2019, 2014) for mouse loci. To increase PCR specificity, nested forward primers were used for the PentaE locus, and only the internal forward primer has the adapter sequence required for NGS. All primer sequences are listed in Tables S1 and S2.

### Optimized PCR amplification and processing using Illumina chemistry

A two-step PCR strategy was used to amplify each STR locus for NGS. PCR1 amplified the individual STR locus and added a partial Illumina adapter. A portion of the PCR1 amplicon was then used as template for PCR2 and added a unique index and the remaining Illumina adaptor to each sample. PCR conditions for STR loci (PCR1) were optimized. Results for detailed optimizations are found in Fig. S1. TMA oxalate solution was made by mixing a 2:1 molar ratio of aqueous TMA hydroxide (Sigma-Aldrich) and ammonium oxalate monohydrate (Thermo Fisher Scientific), respectively (Markowitz, 1957). PCR reactions contained 0.5 mM of each primer, 70 ng genomic DNA, 2 mM TMA oxalate and 1× Platinum SuperFi PCR Master Mix (Thermo Fisher Scientific). The samples were amplified using a Veriti thermal cycler (Thermo Fisher Scientific) as follows: 98°C for 2 min; 28 cycles of 98°C for 15 s, 60°C for 15 s, and then 68°C for 30 s; followed by an extension for 2 min at 72°C. For multiplex PCRs, samples were amplified using the following reaction mixture: 0.2 µM final concentration of primer sets for each locus (six sets in each reaction), 140 ng genomic DNA in the reaction and 1× Platinum SuperFi PCR Master Mix (Thermo Fisher Scientific). Multiplex PCRs were carried out in a Veriti thermal cycler (Thermo Fisher Scientific) with the following cycle conditions: 98°C for 2 min; 30 cycles of 98°C for 15 s, 60°C for 45 s, and then 68°C for 1 min; followed by an extension for 5 min at 72°C. Products of PCR1 were subjected to an additional five cycles with non-overlapping dual-indexing primers without TMA oxalate, and products of PCR2 were pooled and purified using 0.6× SPRI beads (Beckman Coulter). The purified library was run on a BioAnalyzer (Agilent) using a high-sensitivity chip to confirm quality and quantity. It was then denatured with 0.1 nM NaOH, followed by neutralization with 0.1 M HCl. The denatured pool was diluted to 20 pM following Illumina guidelines. It was sequenced on a MiSeqv2 using a MiSeq Reagent Kit 500v2 kit with the following inputs: 250 bp Read1, 10 bp Index1, 8 bp Index2 and 250 bp Read2. Samples were demultiplexed using the index sequences, and FASTQ files were generated using the Illumina bcl2fastq software, allowing 1 bp mismatch.

### STR data analysis

Human STR-CE profiling was performed by Cell Line Genetics (Madison, WI, USA). STR-NGS was analyzed using the python script STRight. This program is a modified version of CRIS.py (Connelly and Pruett-Miller, 2019) and takes a csv file containing target STR data as input. The csv files (STR_human.csv and STR_mouse.csv) contain nine columns: (1) STR name, (2) reference sequence, (3) start target sequence, (4) end target sequence, (5) repeat size, (6) bp_modifier, (7) the number of repeats in a reference sequence found on strbase.nist.gov (this can be used as a quality control check if novel STRs are being added), (8) SNP_modifier and (9) notes. Briefly, the program runs through each line of the fastq files and tests whether the start and end target sequences of each STR can be matched to the read. If both sequences are found, the distance between the two sequences is measured in base pairs. Because SNPs could occur in the flank region outside the STR repeat region, the start and end sequences for STRight analysis do not always land precisely at the beginning and end of each locus. Instead, they may be placed several base pairs upstream or downstream of the repeat to avoid known SNPs. In addition, some STR loci have additional nucleotides inside the repeats (i.e. DS21S11 has 13 additional nucleotides among ‘TCTA’ and ‘TCTG’ repeats). To take these extra base pairs into account, the bp_modifier value is used to subtract any extra base pairs from the total length (i.e. if the start sequence lands 5 bp upstream from the start of the STR repeat region, then a bp_modifier of 5 would subtract 5 bp from the measured distance). The repeat number of a locus is determined by dividing the calculated distance by the size of each repeat, for example, 4 for an ‘AAAG’ repeat. Finally, there are special cases in which SNPs in a given locus create an extra repeat. For example, the STR D13S317 is a ‘TATC’ repeat, which usually terminates right before the sequence ‘AATCAATCATC’. Some individuals, however, have an A>T variant at the first position creating a termination sequence of ‘TATCAATCATC’, which also adds an additional ‘TATC’ repeat (underlining marks the 4 bp region that creates the additional TATC repeat in individuals with the A>T variant). Typical STR-CE utilizes a primer mixture containing two reverse primers that have the 5′ nucleotide landing on either the ‘A’ or ‘T’ of the SNP to detect that variant. STRight contains a SNP_modifier, which checks whether the A/T SNP is present in the read. If the indicated SNP (in this case, ‘T’) is present, then the SNP_modifier value (in this case, 1), would add an extra repeat to the total number to account for the SNP. STRight reports the top repeat lengths for each STR along with sequence information. STRight is free to use, and source code is available at https://github.com/patrickc01/STRight. For STR-NGS data analysis, we define allele percentage as the percentage of reads containing the predominant STR repeat length, stutter as the percentage of reads that contain a repeat length that does not match the predominant STR length and that occurs at a frequency <10%, and noise as the percentage of reads that have partial repeat lengths and that likely occurred through sequencing error or off-target amplification (i.e. 11.2).

### Analysis of mixed samples

Genomic DNA was extracted from diploid iPSC cell lines, BJFF.6 and AN1.1 cells, and added to individual reactions with a final concentration of 140 ng total DNA at BJFF.6/AN1.1 ratios of 1:1, 5:1, 10:1, 20:1, 100:1, 200:1 and 1000:1. PCR amplification is described above, and analysis was performed in the same way as for the single source samples. STR-CE analysis was performed by the St. Jude Hartwell Center for Genome Sequencing using PowerPlex Fusion (Promega).

## Supplementary Material

10.1242/dmm.050150_sup1 Supplementary information
