# Supplementary material for: Short tandem repeat profiling via next-generation sequencing for cell line authentication
Source: Dis Model Mech. 2023 Oct 23;16(10):dmm050150. doi: 10.1242/dmm.050150 (PMC10618599; doi:10.1242/dmm.050150)
Supplement: Supplementary information [file dmm-16-050150-s1.pdf]

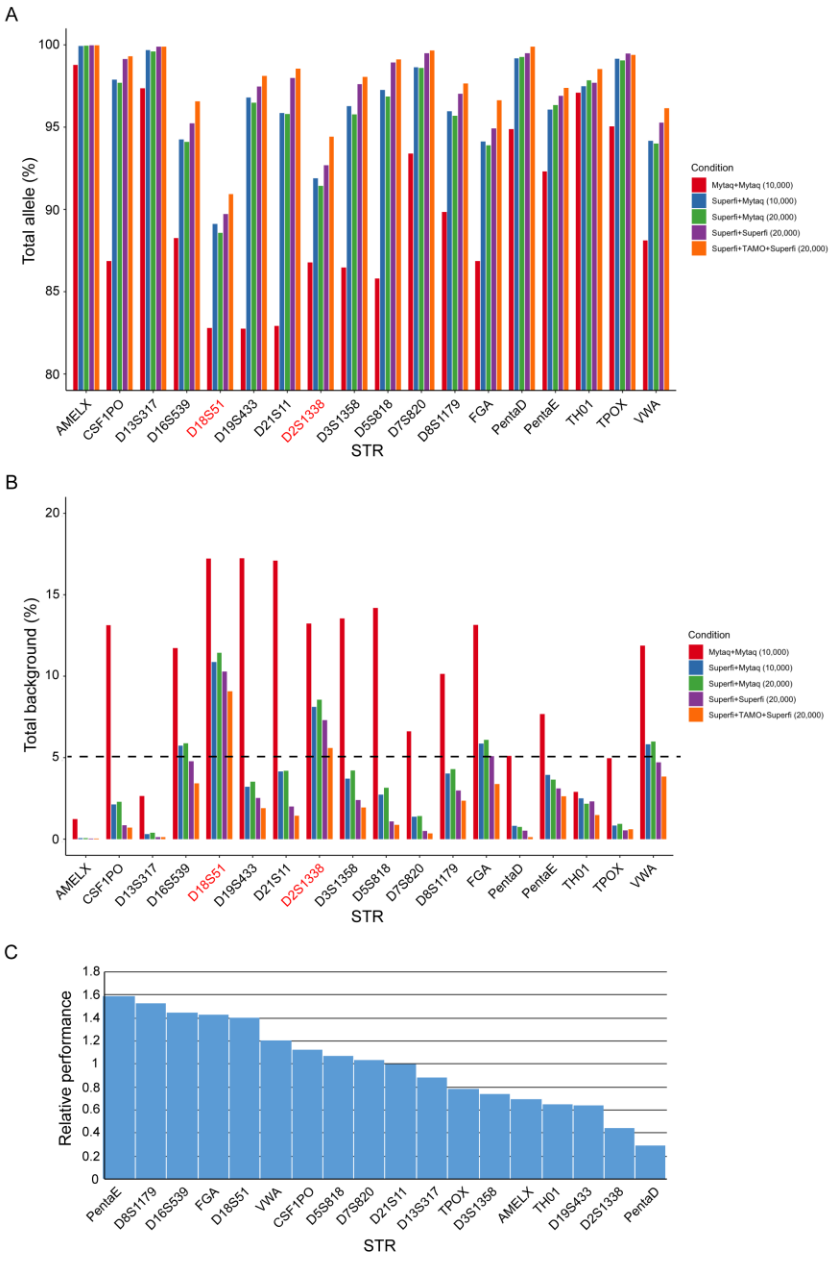

Fig. S1. Human STR loci. (A) Percent of correct call on STR repeat length in each STR locus among different PCR conditions. Figure legend defines the polymerase used for each step of the NGS library construction (PCR1 and PCR2). The number of input cells is shown in parenthesis. (B) Percent of total background reads including stutter and noise in each STR locus among different PCR conditions. Underperforming STR loci with >5% background using the optimized PCR conditions are highlighted in red. (C) Relative STR locus performance (locus specific reads vs. mean of total reads).

Development • Supplementary information

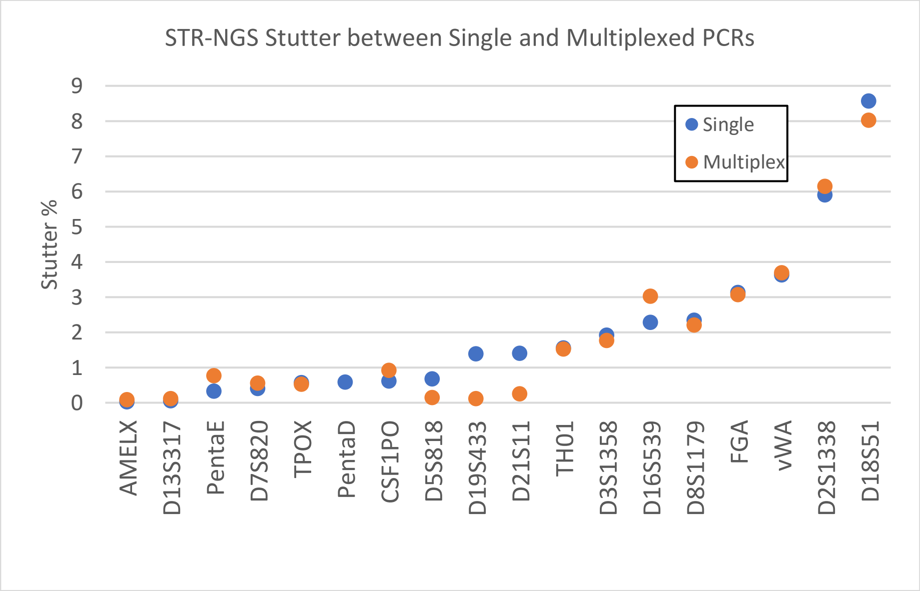

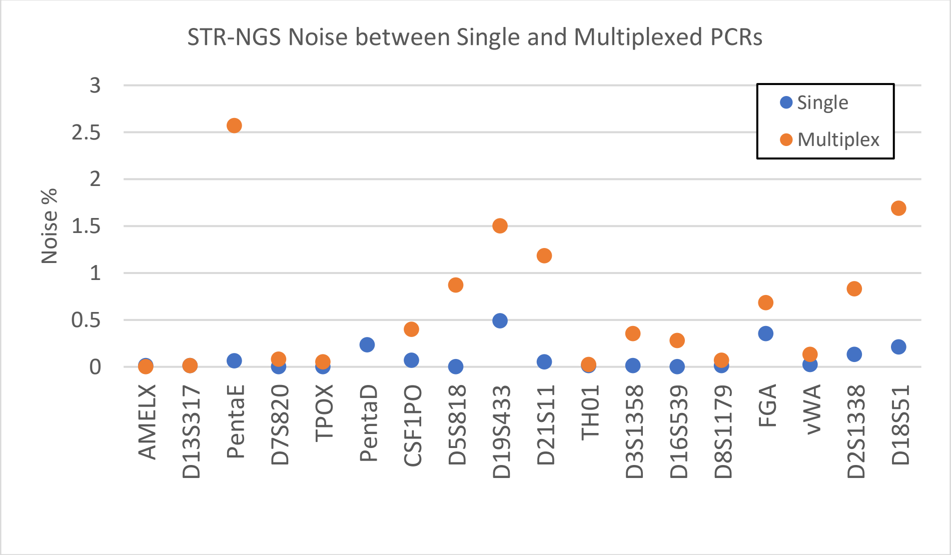

Fig. S2. Comparison of stutter and noise between single and multiplexed PCRs. Noise and stutter are plotted, showing on average less than 1% variance between single and multiplexed PCRs.

Development • Supplementary information

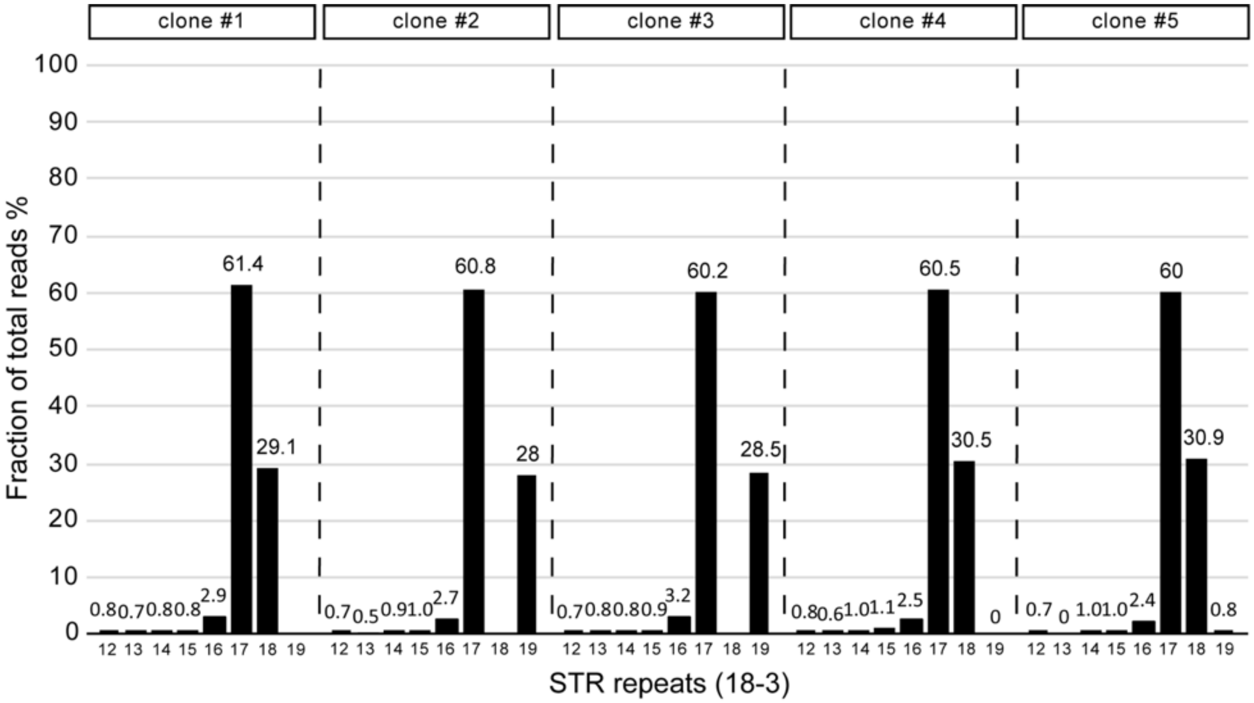

Fig. S3. 18-3 STR locus in NIH3T3 cells. Bar graph displays the percent of each STR repeat length of 18-3 locus in single cell clones of NIH3T3 cell line.

Development • Supplementary information

A

STR repeat	4-2	5-5	6-7	9-2	12-1	15-3	18-3	X-1	1-1	2-1	3-2	8-1	11-2	17-2	19-2
Clone #1	19.3, 20.3	14, 15	12	15, 16	20	20.3	17, 19	25	10	9	14, 15	15	15, 17	13, 14	11, 12
Clone #2	19.3, 20.3	14, 15	12	15, 16	20	20.3	17, 19	25	10	9	14, 15	15	15, 16, 17	13, 14	11, 12
Clone #3	19.3, 20.3	14, 15	12	15, 16	20	20.3	17, 19	25	10	9	14, 15	15	14, 15, 17	13, 14	11, 12

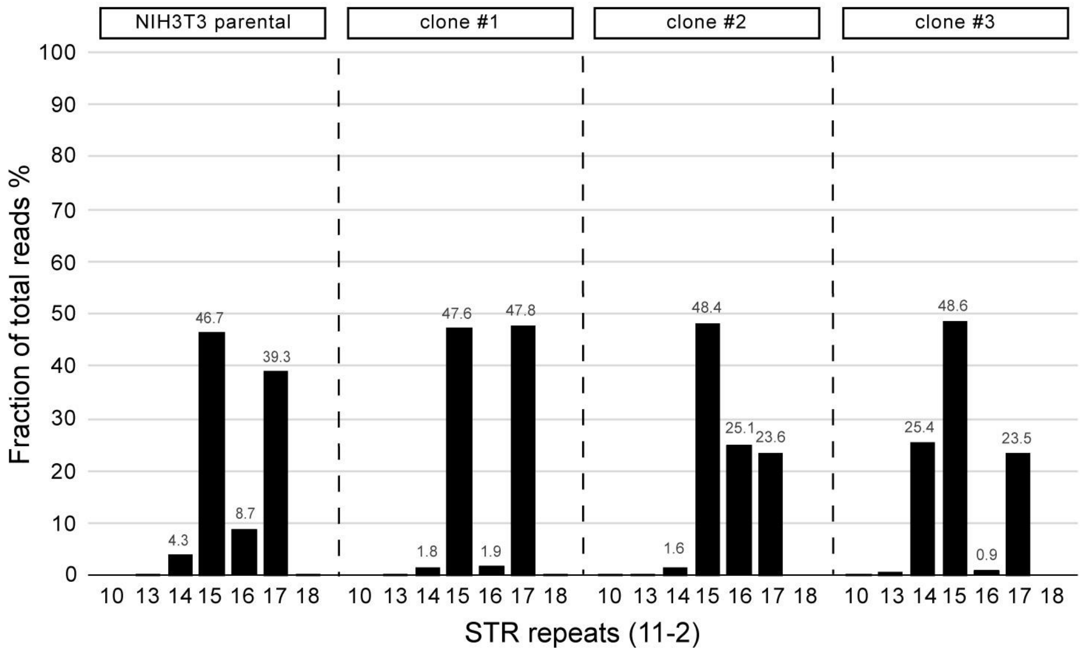

B

STR	STR_CE	STR_NGS_#1	STR_NGS_#2	STR_NGS_#3
11-2	15, 17	15, 17	15, 16, 17	14, 15, 17
Percentage	-	58% (7/12)	25% (3/12)	16.6% (2/12)

Fig. S4. 11-2 STR loci in NIH3T3 cells. (A) Table reports STR repeat lengths of 15 mouse STR loci in single cell clones representing three different subpopulations from NIH3T3 cells. Bar graph demonstrates the percent of each STR repeat length of 11-2 locus in three single cell clones and parental cells. (B) Proportion of three different populations reported for STR repeat at 11-2 locus.

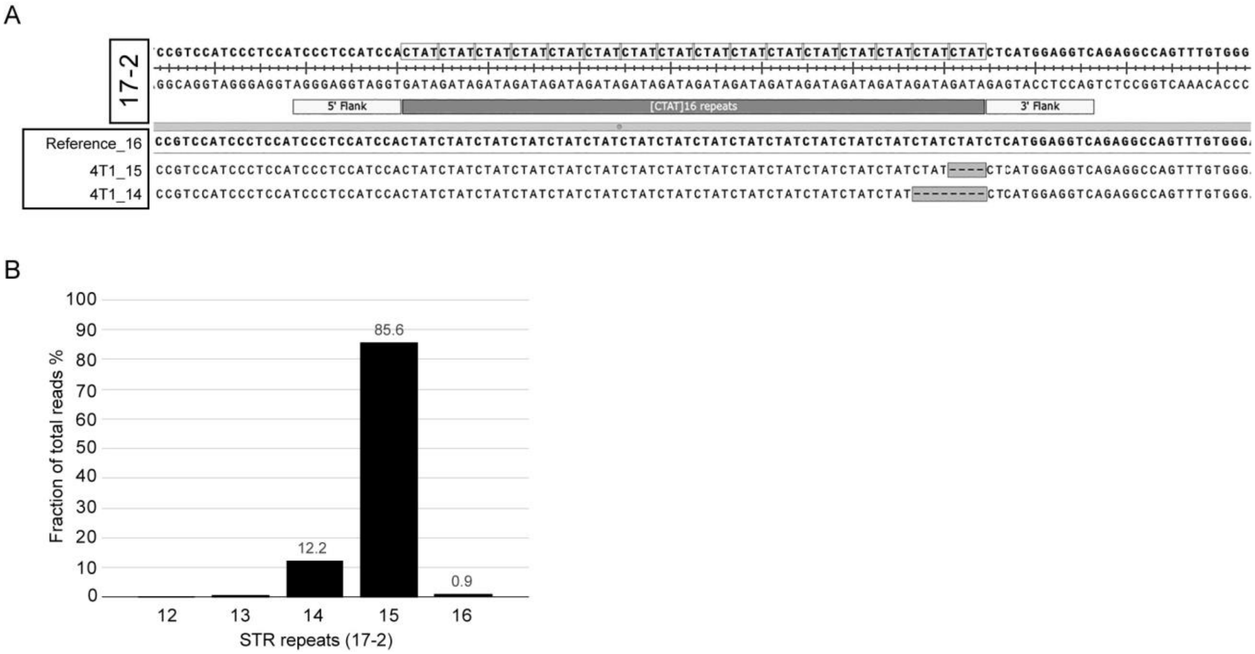

Fig. S5. Sequencing alignment of 17-2 STR locus. (A) Sequence alignments of representative NGS reads among different STR repeat lengths for 17-2 STR locus of 4T1 cell line. (B) Percentage of each STR repeat length in 17-2 locus 4T1 cell line.

Development • Supplementary information

Table S1. Human STR primer sets.

STR locus	Primer Name	Primer Sequence	Amplicon size (bp)
AMELX/Y	AMELX-Y.DS.F	Adapter1 + ACACAGGCTTGAGGCCAAC	~ 217
	AMELX-Y.DS.R	Adapter2 + ATCCTGGGCACCCTGGTTAT	
CSF1PO	CSF1PO.DS.F	Adapter1 + TGAGTCTGCCAAGGACTAGC	~304
	CSF1PO.DS.R	Adapter2 + CACCACTGGCCATCTTC	
D3S1358	D3S1358.DS.F	Adapter1 + GCTTGAGCCCAGGAGTTTGA	~282
	D3S1358.DS.R	Adapter2 + ATGGGCATGCTGGCCATATT	
D5S818	D5S818.DS.F	Adapter1 + GCTTCTAATTAAAGTGGTGTCCC	~255
	D5S818.DS.R	Adapter2 + TCTCAGAGGAATGCTTTAGTGCT	
D8S1179	D8S1179.DS.F	Adapter1 + GCCAGAAACCTCTGTAGCCA	~335
	D8S1179.DS.R	Adapter2 + ACCGTATGTATTCTTGTTTCCAGT	
D13S317	D13S317.DS.F	Adapter1 + GATGGGTTGCTGGACATGGTA	~265
	D13S317.DS.R	Adapter2 + AGAGAGCTTGAATTGTTGGTCAAA	
D21S11	D21S11.DS.F	Adapter1 + ATTCTTCAGCTTGTAGATGGTCTG	~268
	D21S11.DS.R	Adapter2 + AGTCAATGTTCTCCAGAGACAGAC	
FGA	FGA.DS.F	Adapter1 + AAGGCTGCAGGGCATAACAT	~299
	FGA.DS.R	Adapter2 + CCTCTGACACTCGGTTGTAGG	
TH01	TH01.DS.F	Adapter1 + TCTAGCAGCAGCTCATGGTG	~282
	TH01.DS.R	Adapter2 + AAGGTTCTGAGTGCCCAAGG	
TPOX	TPOX.DS.F	Adapter1 + ACTGGCACAGAACAGGCACTTAGG	~244
	TPOX.DS.R	Adapter2 + GGAGGAACTGGGAACCACACAGGT	
VWA	VWA.DS.F	Adapter1 + GCTGAGATGTGAAAGCCCTA	~326
	VWA.DS.R	Adapter2 + ACTAGATACAATAGAGATAGATAGATAGAT	
D2S1338	D2S1338.DS.F	Adapter1 + GGCAATTCCTACTGGCCCAT	~284
	D2S1338.DS.R	Adapter2 + CAGAATGCCAGTCCCAGAGG	
D7S820	D7S820.DS.F	Adapter1 + GAGACGGGGTTTCACCATGT	~256
	D7S820.DS.R	Adapter2 + TCCACATTTATCCTCATTGACAGA	
D16S539	D16S539.DS.F	Adapter1 + GGGGGTCTAAGAGCTTGTAAAAAG	~340
	D16S539.DS.R	Adapter2 + AAAGTAGGTGGTAAAACAGCCT	
D18S51	D18S51.DS.F	Adapter1 + GGAGGAGTTCTTGAGCCCAG	~306
	D18S51.DS.R	Adapter2 + CGTCAGCCTAAGGTGGACAT	
D19S433	D19S433.DS.F	Adapter1 + GTTGAGGCTGCAAAAAGCTATAA	~326
	D19S433.DS.R	Adapter2 + GGGTTCTAGGAATCAATCTTCCT	
PentaE*	PentaE.F	ACCAACATGAAAGGGTACCAATA	~474/342
	PentaE.DS.F	Adapter1 + ACATGGTGAAACCCCGTCTC	
	PentaE.DS.R	Adapter2 + TACTCATTACCTTGCATGCATG	
PentaD	PentaD.DS.F	Adapter1 + GAAGGTCGAAGCTGAAGTGA	~332
	PentaD.DS.R	Adapter2 + GGTATCTTTTGAAGAGTAGAACT	

Development • Supplementary information

Table S2. Mouse STR primer set.

STR locus	Primer Name	Primer Sequence	Amplicon size (bp)
4-2	4-2.DS.F	Adapter1 + AAGCTTCTCTGGCCATTTGA	~ 237
	4-2.DS.R	Adapter2 + TTCATAAACTTCAAGCAATGACA	
5-5	5-5.DS.F	Adapter1 + CGTTTTACCTGGCTGACACA	~341
	5-5.DS.R	Adapter2 + GGTTTAAAACTCAATACCAAACAA	
6-7	6-7.DS.F	Adapter1 + AGTCCACCCAGTGCATTCTC	~278
	6-7.DS.R	Adapter2 + GGCTTTAATTCCCCAGAATCTGT	
9-2	9-2.DS.F	Adapter1 + GGATTGCCAAGAATTTGAGG	~228
	9-2.DS.R	Adapter2 + TCCTGAGTTGTGGACAGGGTTA	
12-1	12-1.DS.F	Adapter1 + AGCTACGTAAATATGAGAGGGTTT	~325
	12-1.DS.R	Adapter2 + ATCAGAATGAACCAATGGTCAAGA	
15-3	15-3.DS.F	Adapter1 + TCTGGGCGTGTCTGTCATAA	~196
	15-3.DS.R	Adapter2 + TTCTCAGGGAGGAGTGTGCT	
18-3	18-3.DS.F	Adapter1 + CAAGGGCCAGACTTGGACAATA	~285
	18-3.DS.R	Adapter2 + TAGCTCAGGGGTAGAGAGTATGC	
X-1	X-1.DS.F	Adapter1 + GGGAGCATCACCTCTCAAAGATA	~363
	X-1.DS.R	Adapter2 + ATGGGATTGTGTGGTATCCTACT	
1-1	1-1.DS.F	Adapter1 + CCCCTTCACTCCTTCATTCCA	~355
	1-1.DS.R	Adapter2 + CTCCCTTGCTGTACACTCCG	
2-1	2-1.DS.F	Adapter1 + AGTCTTTGTGGCTGGACCGAG	~259
	2-1.DS.R	Adapter2 + GAGCAATCACTGGCCTTGGTAG	
3-2	3-2.DS.F	Adapter1 + AAGCTGTAACATATTTGTCTGTC	~301
	3-2.DS.R	Adapter2 + GACATGTGATAGAGGATGGATAG	
8-1	8-1.DS.F	Adapter1 + ATGAAGCCCCAAGCTCCATC	~291
	8-1.DS.R	Adapter2 + GCCTAGAACTGGGCCTGG	
11-2	11-2.DS.F	Adapter1 + GGTGGCTCACAACCATCAGT	~255
	11-2.DS.R	Adapter2 + ATCCACGTCTTGAGGGCTG	
17-2	17-2.DS.F	Adapter1 + TGTGCTCCAGAACCATCCTG	~305
	17-2.DS.R	Adapter2 + AGCACAAACTGAGAGAGCCT	
19-2	19-2.DS.F	Adapter1 + TTGGTCTGGTTAGTTCCCTGG	~296
	19-2.DS.R	Adapter2 + AATGGATTTAAAAAGTGAGGTTCCC	

Development • Supplementary information

Table S3. STR-CE PowePlex Fusion analysis of mixed samples. gDNA from BJFF and AN1 cells was mixed at defined ratios and analyzed using STR-CE. Limit of detection for AN1 gDNA was at a ratio of 1:5.

User	ng/µl	Comment(s)
Connelly	7.49	A full profile was obtained.
Connelly	6.61	A full profile was obtained.
Connelly	6.08	This sample is contaminated and contains at least 2 sources of DNA.
Connelly	8.71	This sample is contaminated and contains at least 2 sources of DNA.
Connelly	1.76	A full profile was obtained.
Connelly	5.30	A full profile was obtained.
Connelly	6.95	A full profile was obtained.
Connelly	7.49	A full profile was obtained.

Development • Supplementary information
